# An Inexpensive Method for Kinematic Calibration of a Parallel Robot by Using One Hand-Held Camera as Main Sensor

**DOI:** 10.3390/s130809941

**Published:** 2013-08-05

**Authors:** Alberto Traslosheros, José María Sebastián, Jesús Torrijos, Ricardo Carelli, Eduardo Castillo

**Affiliations:** 1 Postgrado, Universidad Aeronáutica de Querétaro, Carretera Estatal 200, Tequisquiapan 22154, 76270 Colón, Mexico; E-Mail: altrami@hotmail.com; 2 CAR-UPM-CSIC, Universidad Politécnica de Madrid, c/José Gutiérrez Abascal 2, 28006 Madrid, Spain; E-Mail: jesus.torrijos.ramirez@alumnos.upm.es; 3 Instituto de Automática, Universidad Nacional de San Juan, Av. San Martín Oeste 1109, 5400 San Juan, Argentina; E-Mail: rcarelli@inaut.unsj.edu.ar; 4 Instituto Politécnico Nacional, CICATA, México, Department of Mechatronics, Cerro Blanco 141, 76090 Querétaro, Mexico; E-Mail: ecastilloca@ipn.mx

**Keywords:** kinematic calibration, external sensor calibration, one-camera-in-hand sensor, parallel mechanism, parallel robot, visual servoing

## Abstract

This paper presents a novel method for the calibration of a parallel robot, which allows a more accurate configuration instead of a configuration based on nominal parameters. It is used, as the main sensor with one camera installed in the robot hand that determines the relative position of the robot with respect to a spherical object fixed in the working area of the robot. The positions of the end effector are related to the incremental positions of resolvers of the robot motors. A kinematic model of the robot is used to find a new group of parameters, which minimizes errors in the kinematic equations. Additionally, properties of the spherical object and intrinsic camera parameters are utilized to model the projection of the object in the image and thereby improve spatial measurements. Finally, several working tests, static and tracking tests are executed in order to verify how the robotic system behaviour improves by using calibrated parameters against nominal parameters. In order to emphasize that, this proposed new method uses neither external nor expensive sensor. That is why new robots are useful in teaching and research activities.

## 1. Introduction

Parallel robots or parallel kinematic machines (PKM), in contrast to serial robots, are characterized by high structural stiffness, high load operation, high speed and acceleration of the end effector and high accuracy positioning of the end effector. This is why parallel robots are commonly used in tasks such as surgery, material machining, electronics manufacturing, and product assembly among many other applications. In order to reach such high robot accuracy, it is necessary to avail robust and reliable calibration methods, which is quite difficult to obtain from both theoretical and practical point of view, even if it could be performed off-line. Robot accuracy can be affected by increasing backlash due to robot operation, thermal effects, robot control, robot dynamics [1], manufacturing errors and element deformation [2,3]. Nowadays, there are many robot calibration methods, so it is not easy to provide a good classification in order to know the advantages and disadvantages of each one. Furthermore, there are even some hybrid calibration methods. However, in general, it is possible to classify different calibration strategies in three main groups by considering the location of the measurement instruments and their additional elements (for a good overview, see [4]). Thus, there are external, constrained and auto calibration methods. *External calibration* is based on measurements of several poses (if orientation is considered) of the robot end effector (or other structural element) by using an external instrument. *Constrained calibration* methods rely on mechanical elements to constraint some kind of motion of the robot during the calibration process. This method is generally simple and it is considered the most inexpensive. And finally, *auto calibration* methods are those that calibrate the robot automatically, even during the robot operation [2,5–7]. In general auto (or self) calibration methods are more expensive due to their complexity. They are even used for including redundant sensors [8], redundant elements or more complex algorithms. The calibration method shown in this article is considered an external calibration method.

As previously mentioned, external calibration can generally be carried out by measuring pose parameters of the end effector of the robot (or other elements of the robot) completely or partially. Measurements of the pose of a platform can be made with a laser [9] and a coordinate measuring machine (CMM) [2,10], commercial visual systems [11], laser sensor [12], including additional passive legs [13], interferometers [14], LVDT and inclinometers in [15,16], theodolite [17], gauges [18], double ball bar (DBB) [19], inspection of a machine part that is dedicated to the calibration process [2,20], accelerometers [21], or visual systems and patterns that have been widely studied (*i.e.*, chess-board) [6,22–25]. The above examples represent different strategies that can be used in order to obtain kinematic information about the robot, but in general, calibration methods impose virtual or real constraints on the poses of the end effector (or mobile elements). By choosing an appropriate method, a calibration can be an economical and practical technique in order to improve accuracy of a parallel robot (PKM) [25].

Generally, the goal of a kinematic calibration process is to obtain the constraints of quite large measured poses (called calibration poses) in order to determine which geometry (distances or angles of elements) of the robot is the best to satisfy the above constraints [26]. It should be mentioned that there are more recent approaches, which can be identify both model structure and parameters [27], obtain a range of solutions (interval analysis) that guarantee parameter values to satisfy the calibration equations or approaches that obtain a few, but high accuracy poses [9]. The above basic idea has been applied to the calibration of serial and parallel robots. The main differences are found in the measurement instruments and strategies. Visual methods are becoming more popular due to their simplicity and low cost compared to other methods. Parallel robots visual techniques were first proposed by Amirat [28]. Stereo visual methods are developed [29,30] but other visual methods based on a monocular system [6,22–25,31,32] propose using a pattern or marks on a flat surface. These kinds of patterns are employed in camera calibration where intrinsic and extrinsic parameters [33] can be obtained. By means of the extrinsic parameters of the camera it is possible (by attaching the camera or the pattern to the end effector of the robot) to obtain the pose of the end effector of the robot and consequently consider the poses as calibration poses.

In this paper a novel, inexpensive, and external calibration method to calibrate a parallel robot with three DOF is proposed. In order to obtain the joint and 3D incremental positions, the proposed method utilizes positions (not absolute but incremental positions) from the resolvers of the motors and visual information obtained from a spherical element. Once the above information is obtained, it is used in the constraint equations to solve them numerically and obtain the best set of geometrical parameters that will satisfy the set of equations. Finally, several tests are executed in order to check the best behaviour with calibrated parameters against nominal parameters.

The main contribution of this approach is the use of low-cost sensor for calibrating the robot and the methodology used with the approval of the obtained results. Also it allows the evaluation of the position accuracy of homemade robot and the easy application to other robots developed in educational and research teams.

## 2. System Description

**Figure 1.**
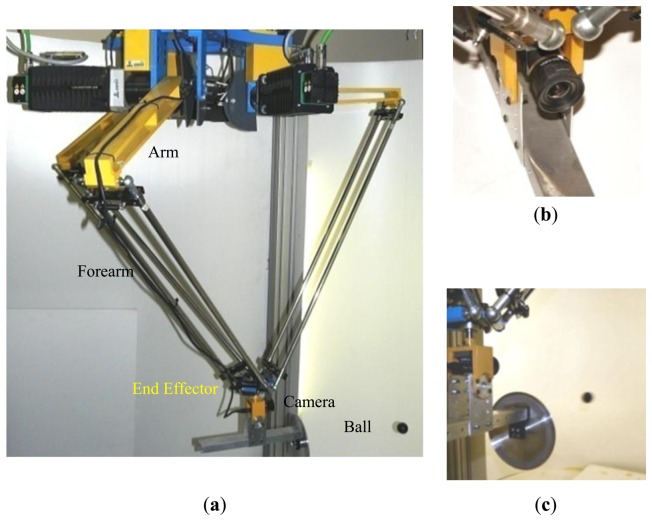
**(a)** System RoboTenis, **(b)** Robot camera, **(c)** Robot environment.

The main objective of the proposed method is to calibrate a parallel robot of the system called RoboTenis. The calibration of the robot is used for improving accuracy of its visual servoing algorithms and developing future tasks such as playing a game of ping-pong. The RoboTenis system was created to study and design visual servoing controllers and to carry out object tracking under dynamic environments. The mechanical structure of the RoboTenis system was inspired by the DELTA robot [34,35]. Its vision system is based on one camera allocated at the end effector of the robot. Basically, the RoboTenis platform (Figure 1a) consists of a parallel robot and a visual system for acquisition and analysis. The maximum end effector speed of the parallel robot is 6 m/s and its maximum acceleration is 58 m/s**^2^**. The visual system of the platform RoboTenis [36] is made up of a 0.05 kg camera that is located at the end effector (Figure 1b,c) and a frame grabber (SONY XCHR50 and Matrox Meteor 2-MC/4 respectively). The motion system is composed of three AC brushless servomotors, Ac drivers (Unidrive), and planetary gearboxes. The joint controller is implemented in a DS1103 card (software implemented in ANSI C). The camera has been calibrated following the method proposed by Zhan [32].

## 3. Constraint Equations

**Figure 2.**
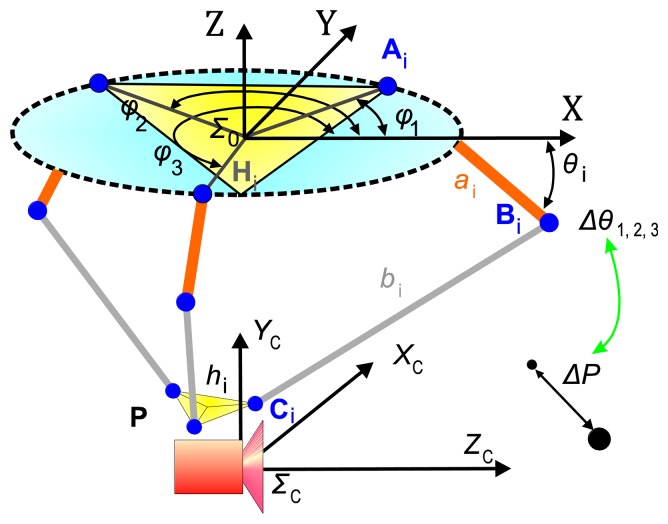
A sketch of the RoboTenis system.

Constraint equations for the calibration method were obtained from a kinematic model of the robot. It is important to emphasize that the transformation matrix, between the camera coordinates system and the effector coordinates system, does not intervene in the equations of constraint used in the calibration of the robot, but it is obtained during the camera calibration process. Transformation matrix was divided into rotation and translation components. The rotation matrix was obtained independently from the robot calibration, whereas translation matrix is not necessary since the camera is fixed to the end effector and the model of the constraint equations is incremental. Figure 2 shows a sketch of the parallel robot, where:
***Σ****_o_*is the robot reference frame*i*is the forearm and arm number, (*i ∈ {1,2,3};**k*is the incremental measurement number, *(k ∈ N;****A****_i_*is the joint robot base arm, revolute axis of motor *i****B****_i_*is the joint arm and forearm *i*, ***B****_i_* = [*B_x_B_y_B_z_*]**^T^***_i_****C****_i_*is the joint effector forearm *i*, ***C****_i_* = [*C_x_C_y_C_z_*] **^T^***_i_****P***is the centre of the end effector, ***P*** = [*P_x_P_y_P_z_*] **^T^*****P****_0_*is the initial end effector position, ***P****_0_* = [*P_0x_P_0y_P_0z_*] **^T^***a_i_*is the arm length for the joint *i**b_i_*is the forearm *i* length*h_i_*is the distance from ***P*** to ***C****_i_**H_i_*is the distance from the robot reference frame ***Σ****_o_* to ***A****_i_**θ_i_*is the motor angle*θ_i0_*is the initial motor angle (unknown)*Φ_i_*is the angle in which point ***A****_i_* is allocated in the *XY* plane of the robot reference frame

**Figure 3.**
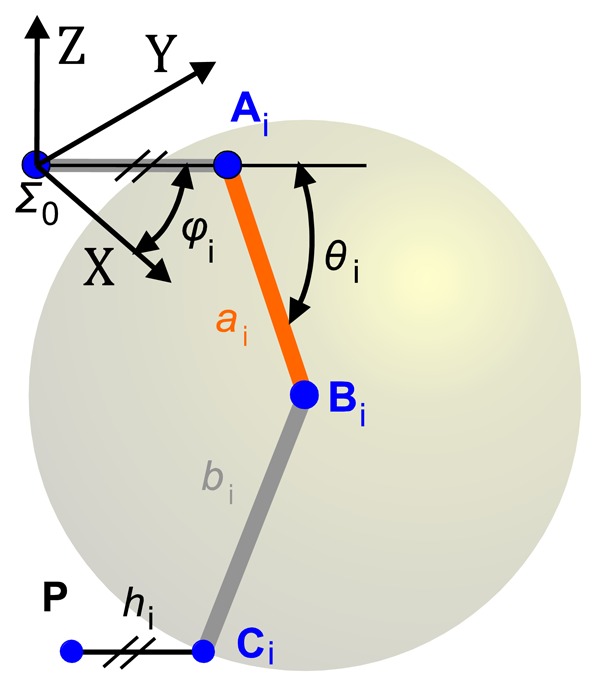
One leg profile of the RoboTenis system.

The constraint equations are based on the movement of the forearms with respect to the robot arms [37]. In Delta robot, the forearms are parallelograms that link the end effector to the arms by means of spherical joints. Spherical joints allow modelling the movement of the end effector as the intersection of three spherical surfaces that are described by points ***C****_i_* in Figure 3. By inspecting Figure 2 and Figure 3, it is possible to obtain the surface of a sphere described by the point ***C****_i_* with centre in ***B****_i_* as:

(1) $$\Gamma_{i}={\left(C_{ix}-B_{ix}\right)}^{2}+{\left(C_{iy}-B_{iy}\right)}^{2}+{\left(C_{iz}-B_{iz}\right)}^{2}-b_{i}^{2}=0$$

where ***B****_i_* and ***C****_i_* can be expressed in the robot frame, ***Σ****_o_* as:

(2) $$\begin{array}{c} \left[\begin{array}{c} B_{x}\\ B_{y}\\ B_{z} \end{array}\right]=\left[\begin{array}{c} (H+a\;\cos\theta )\cos\phi \\ (H+a\;\cos\theta )\sin\phi \\ a\;\sin\theta \end{array}\right]\\ \left[\begin{array}{c} C_{x}\\ C_{y}\\ C_{z} \end{array}\right]=\left[\begin{array}{c} P_{x}\\ P_{y}\\ P_{z} \end{array}\right]-\left[\begin{array}{ccc} \cos\phi & -\sin\phi & 0\\ \sin\phi & \cos\phi & 0\\ 0 & 0 & 1 \end{array}\right]\;\left[\begin{array}{c} -h\\ 0\\ 0 \end{array}\right] \end{array}$$

Since incremental joint and incremental end effector positions can be obtained (a similar approach for serial robots can be found in [38]), each absolute measurement can be expressed as:

(3) $$\begin{array}{ccc} \theta_{ik}=\theta_{i0}+\Theta_{ik} & \text{and} & P_{k}=P_{0}+\Pi_{k} \end{array}$$

Observe that *θ_i0_* and ***P****_0_* are unknown but *Θ_ik_* = *Δθ_ik_* = *θ_ik_* − *θ_i0_* and ***Π****_k_* =*Δ****P****_k_* =***P****_k_* − ***P****_0_* are incremental positions and measured by the resolvers and the camera. So ***V*_k_** = [*Θ_1_Θ_2_Θ_3_Π_x_Π_y_Π_z_*]*_k_* is a vector of measured parameters and it represents incremental positions of the end effector; they are measured from sphere images and *k* is the incremental measurement number.

## 4. Solution of the Constraints Equations

Not all of the robot parameters have an effect on the kinematic model and as a consequence they cannot be identified. This fact is known as observability of the term. An observability measurement can be derived from the Jacobian of the constraint equations. This Jacobian is called observation matrix and observable parameters can be obtained by its **QR** decomposition [39]. In the model, by means of the **QR** decomposition, the influences of the parameters *Φ_i_* and *H_i_* are difficult to observe. For this reason they are not identified in this work but the nominal value is taken into account. On the other hand, identifiable parameters are: *a_i_*, *b_i_*, *h_i_*, and the reference positions of the actuators and the end effector of the robot: *θ_i0_* and ***P****_0_* respectively (***P****_0_* is the initial end effector position). Thus the unknown parameters can be expressed as: ***u****_i_* = [*a_i_b_i_h_i_θ_i0_*] and ***U*** = [***u****_1_****u****_2_****u****_3_P_x0_P_y0_P_z0_*]

Substituting the incremental Equations (3) in Equations (2) and (1), the constraint equations can be arranged as:

(4) $$\begin{array}{c} \Gamma_{ik}={\left(P_{x0}+h_{i}\cos\phi_{i}+\Pi_{xk}-(a_{i}\cos(\theta_{i0}+\Theta_{ik})+H_{i})\cos\phi_{i}\right)}^{2}\\ \;+{\left(P_{y0}+h_{i}\text{sin}\phi_{i}+\Pi_{yk}-(a_{i}\cos(\theta_{i0}+\Theta_{ik})+H_{i})\sin\phi_{i}\right)}^{2}\\ \;+{\left(P_{z0}+\Pi_{zk}-a_{i}\cos(\theta_{i0}+\Theta_{ik})\right)}^{2}-b_{i}^{2}=0 \end{array}$$

In order to solve the above equations, Equation (4) is grouped as:

(5) $$\Phi (\text{U},V_{k})={\left[\Gamma_{11}\ldots \Gamma_{1\kappa }\Gamma_{21}\ldots \Gamma_{2\kappa }\Gamma_{31}\ldots \Gamma_{3\kappa }\right]}^{T}$$

Note that because the constraint equations are not linear, their solution is no exactly satisfied, thus, commonly, an approximate solution is obtained by numerical algorithms such as the Gauss-Newton method. Other possible alternatives are shown in [40]. By expressing the constraints in Equation (5) in them Taylor linear approximation (to be solved iteratively):

(6) $$\Phi ({\text{U}}_{n+1},{\text{V}}_{k})\approx \Phi ({\text{U}}_{n},V_{k})+J(U_{n},V_{k})(U_{n+1}-U_{n})=0$$

where *n* is the iteration number and ***J***_3k × 15_ is the Jacobian of the constraint equations or the observation matrix, which is given by:

(7) $$\begin{array}{c} \text{J}(U_{n},V_{k})=\left[\begin{array}{c} J_{1k}\\ J_{2k}\\ J_{3k} \end{array}\right]\\ J_{1k}=\left[\begin{array}{cccccccccccc} \frac{\partial \Gamma_{11}}{\partial a_{1}} & \cdots & \frac{\partial \Gamma_{11}}{\partial \theta_{10}} & 0 & \cdots & 0 & 0 & \cdots & 0 & \frac{\partial \Gamma_{11}}{\partial P_{x0}} & \frac{\partial \Gamma_{11}}{\partial P_{y0}} & \frac{\partial \Gamma_{11}}{\partial P_{z0}}\\ \vdots & \vdots & \vdots & \vdots & \vdots & \vdots & \vdots & \vdots & \vdots & \vdots & \vdots & \vdots \\ \frac{\partial \Gamma_{1k}}{\partial a_{1}} & \cdots & \frac{\partial \Gamma_{1k}}{\partial \theta_{10}} & 0 & \cdots & 0 & 0 & \cdots & 0 & \frac{\partial \Gamma_{1k}}{\partial P_{x0}} & \frac{\partial \Gamma_{1k}}{\partial P_{y0}} & \frac{\partial \Gamma_{1k}}{\partial P_{z0}} \end{array}\right]\\ J_{2k}=\left[\begin{array}{cccccccccccc} 0 & \cdots & 0 & \frac{\partial \Gamma_{21}}{\partial a_{2}} & \cdots & \frac{\partial \Gamma_{21}}{\partial \theta_{20}} & 0 & \cdots & 0 & \frac{\partial \Gamma_{21}}{\partial P_{x0}} & \frac{\partial \Gamma_{21}}{\partial P_{y0}} & \frac{\partial \Gamma_{21}}{\partial P_{z0}}\\ \vdots & \vdots & \vdots & \vdots & \vdots & \vdots & \vdots & \vdots & \vdots & \vdots & \vdots & \vdots \\ 0 & \cdots & 0 & \frac{\partial \Gamma_{2k}}{\partial a_{2}} & \cdots & \frac{\partial \Gamma_{2k}}{\partial \theta_{20}} & 0 & \cdots & 0 & \frac{\partial \Gamma_{2k}}{\partial P_{x0}} & \frac{\partial \Gamma_{2k}}{\partial P_{y0}} & \frac{\partial \Gamma_{2k}}{\partial P_{z0}} \end{array}\right]\\ J_{3k}=\left[\begin{array}{cccccccccccc} 0 & \cdots & 0 & 0 & \cdots & 0 & \frac{\partial \Gamma_{31}}{\partial a_{3}} & \cdots & \frac{\partial \Gamma_{31}}{\partial \theta_{30}} & \frac{\partial \Gamma_{31}}{\partial P_{x0}} & \frac{\partial \Gamma_{31}}{\partial P_{y0}} & \frac{\partial \Gamma_{31}}{\partial P_{z0}}\\ \vdots & \vdots & \vdots & \vdots & \vdots & \vdots & \vdots & \vdots & \vdots & \vdots & \vdots & \vdots \\ 0 & \cdots & 0 & 0 & \cdots & 0 & \frac{\partial \Gamma_{3k}}{\partial a_{3}} & \cdots & \frac{\partial \Gamma_{3k}}{\partial \theta_{30}} & \frac{\partial \Gamma_{3k}}{\partial P_{x0}} & \frac{\partial \Gamma_{3k}}{\partial P_{y0}} & \frac{\partial \Gamma_{3k}}{\partial P_{z0}} \end{array}\right] \end{array}$$

Finally the parameters are iteratively obtained by:

(8) $$U_{n+1}=U_{n}-J{\left(U_{n},V_{k}\right)}^{+}\Phi (U_{n},V_{k})=0$$

where, ***J*^+^** is the pseudo inverse Jacobian. Initial values (***U****_0_*) are given by the nominal parameters and a pose of the robot (given from nominal parameters). In the constraint equations in Equation (4), the units of the Jacobian (7) are homogeneous. There is not need to normalize the Jacobian.

## 5. Visual Measurement System

**Figure 4.**
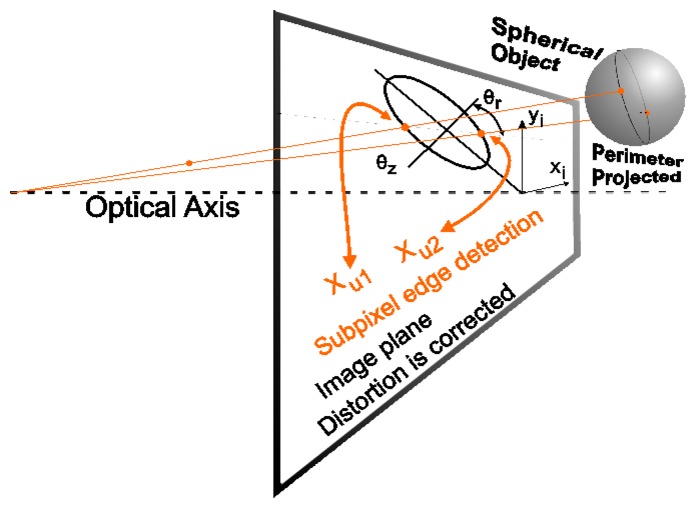
Projection of the ball in the image plane.

The visual measurement system is made up of a fixed (by a transparent rod) ball and a calibrated camera that is located at the end effector of the robot. The visual system has to get the relative robot position with respect to the ball in order to deliver values used in Equation (8) to the joint incremental and the end effector position. In this section, various fundamental points are analyzed to reach a good accuracy in the parameters from the robot calibration. On the one hand, it is necessary to set the wanted end effector positions to their number. On the other hand, it is also necessary to get the visual data with the greatest accuracy as possible. Besides using subpixel information, it has been considered important to correct two ball projection distortions by mean of a novel algorithm. The projected sphere is seen as an ellipse in the image. A first effect to correct is that the ellipse does not match with the projection of a ball section with the real ball centre. The second effect is the difference between the axis ellipse extremes and the values obtained in the horizontal and vertical lines that cross the ellipse centre (see Figure 4).

### 5.1. Selection of Calibration Poses and Obtained Robot Parameters

In order to solve the system of constraint equations, a set of incremental positions (resolvers and end effector) is needed. Several simulations have shown that at least 6 different perfect pose measurements are required. However, it is obvious that perfect measurements cannot be obtained. Therefore, it is necessary to choose best calibration poses. Thus, errors in the parameters of the robot are especially critical when the calibration pose is near a singularity. However in a practical calibration process, if poses are extremely close to singularities, they can produce unpredictable movements (due to kinematic errors). In this work, the robot workspace is numerically well known, by using nominal values, and the calibration poses are chosen randomly from the workspace boundaries. Thus if a pose is located between 5 and 10 cm from a singularity, then it is included as a calibration pose. The experiments were implemented with 300 poses and each measure is the mean of 500 images in order to avoid noise from image acquisition (image acquisition rate: 8.34 ms) 21 minutes are needed for the acquisition of all images. Additionally, it is necessary to wait 4 seconds at each change of pose in order to ensure the absence of any vibration in the robot or the holding structure. The image processing is performed while the acquisition process, while the time taken by the optimization algorithm is almost negligible compared to the rest (about 5 seconds). Due to its simplicity, the calibration process is done automatically in 40 minutes and it can be calibrated on-line. Several calibrations have been executed and then several sets of calibrated parameters have been used in the initial tests. Since similar results have been obtained, the set of calibrated parameters that produces less error in the tests (see Section 6) has been chosen to compare with the nominal parameter in the rest of the tests. In Table 1, both parameter sets are shown.

### 5.2. Three-Dimensional Position Correction

**Figure 5.**
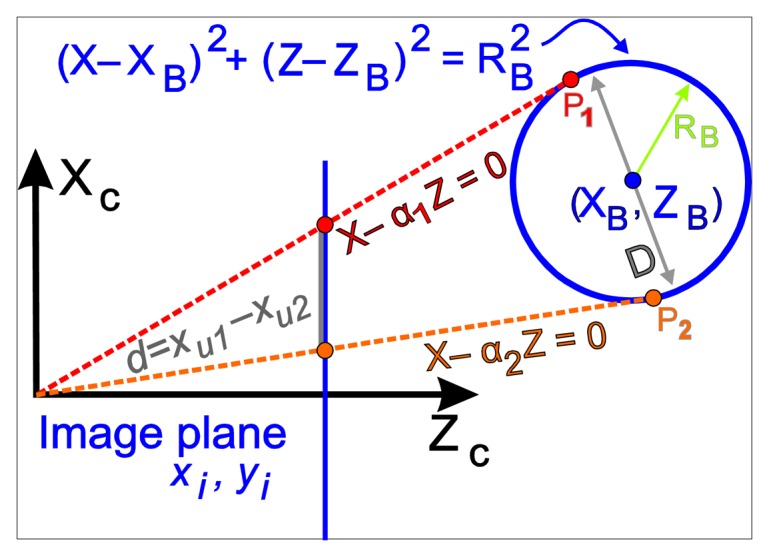
Simplified projection of the ball in the image plane.

As previously mentioned, the aim is to obtain the position of the centre of the ball, (*X_B_*, *Y_B_*, *Z_B_*) shown in the camera coordinates, from the acquired visual information. With the data from the kinematic camera calibration, the relative position between the robot and the fixed ball is determined. In this section, an algorithm that makes a first correction of the ball projection in order to increase the accuracy of the detection is detailed out. The projected sphere is seen as an ellipse in the image. Nevertheless, the ellipse does not match with the projection of a ball section with the real ball centre. This little difference thereby affects the obtained position. In the method description a 2D model as shown in Figure 5 is used. The plane *Xc*-*Zc* (camera reference system) that contains the sphere centre *(Yc* = *Y_B_)* is shown. On the other hand, each point in the image is related by a line that passes through the optical centre of the camera. In this case, the interesting points are the points that belong to the two diametrically opposed tangents to the sphere.

The image points, *x_u1_* and *x_u2_*, in central images coordinates correspond with the projection of the points ***P****_1_* and ***P****_2_*, whose distance is not the ball diameters strictly. So:

(9) $$\begin{array}{ccc} X_{1}=\alpha_{1}Z_{1} & \text{and} & X_{2}=\alpha_{2}Z_{2} \end{array}$$

The values *α_1_* and *α_2_* are obtained from the projection of the tangential points to the sphere, *x_u1_* and *x_u2_*. It will be:

(10) $$\begin{array}{cc} \frac{x_{u1}}{f}=\frac{X_{1}}{Z_{1}}=\alpha_{1}; & \frac{x_{u2}}{f}=\frac{X_{2}}{Z_{2}}=\alpha_{2} \end{array}$$

where, *f* is the focal distance (determined in the camera calibration process). The sphere is fixed in the space, its radio is known (*R_B_* = 19 mm), and the distance of the line that is tangent to the perimeter to the centre of the sphere (*X_B_*, *Y_B_*, *Z_B_*) is given by:

(11) $$R_{B}=\frac{\left|X_{B}-\alpha_{1,2}Z_{B}\right|}{\sqrt{1+\alpha_{1,2}^{2}}}$$

The term (*X_B_*−*α*_1,2_*Z_B_*) has different sign in the extreme values (it is null for values near to the mean value), then following equations are possible:

(12) $$\begin{array}{c} -X_{B}+\alpha_{1}Z_{B}=R_{B}\sqrt{1+\alpha_{1}^{2}}\\ X_{B}-\alpha_{2}Z_{B}=R_{B}\sqrt{1+\alpha_{2}^{2}} \end{array}$$

From here it can obtain *X_B_* and *Z_B_* directly but it is necessary to take the *Y_c_*-*Z_c_* plane information. So, the same way in this plane the tangential points will fulfil:

(13) $$\begin{array}{ccc} Y_{1}=\beta_{1}{\text{Z}}_{1} & \text{and} & Y_{2}=\beta_{2}Z_{2} \end{array}$$

The values of *β_1_* and *β_2_* are got from the projection of the tangential points to the sphere, *y_u1_* and *y_u2_*. It will be:

(14) $$\begin{array}{cc} \frac{y_{u1}}{f}=\frac{Y_{1}}{Z_{1}}=\beta_{1}; & \frac{y_{u2}}{f}=\frac{Y_{2}}{Z_{2}}=\beta_{2} \end{array}$$

Likewise, considering the distance from the sphere centre to each tangent line:

(15) $$R_{B}=\frac{\left|Y_{B}-\beta_{1,2}Z_{B}\right|}{\sqrt{1+\beta_{1,2}^{2}}}$$

With similar reasons that for Equation (12):

(16) $$\begin{array}{c} -Y_{B}+\beta_{1}Z_{B}=R_{B}\sqrt{1+\beta_{1}^{2}}\\ Y_{B}-\beta_{2}Z_{B}=R_{B}\sqrt{1+\beta_{2}^{2}} \end{array}$$

Then, with Equations (12) and (16) there are four equations with three unknowns, (*X_B_*, *Y_B_*, *Z_B_*), that can be solved using the least squares method.

### 5.3. Ellipse Ball Projection Shape Correction

**Figure 6.**
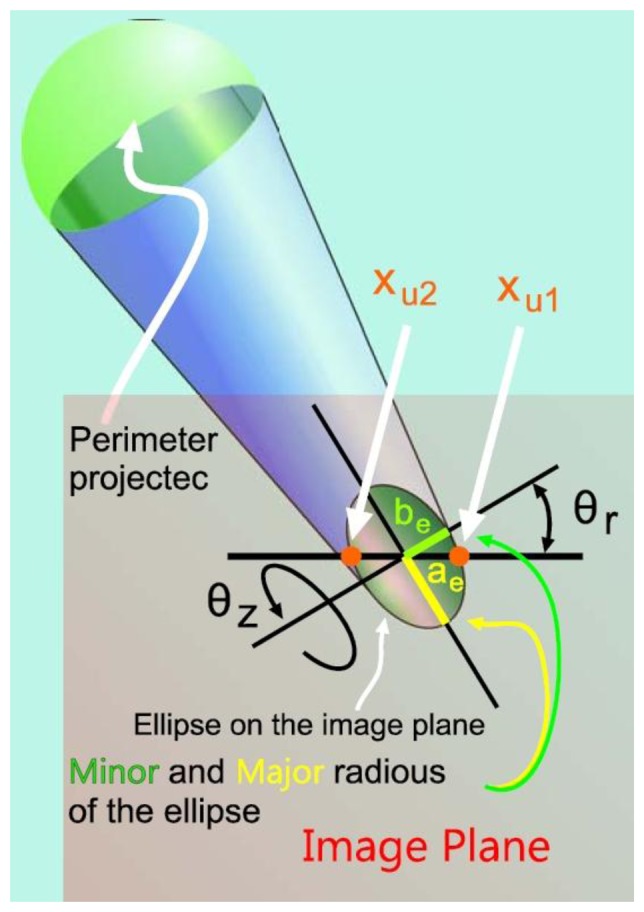
Ellipse of the sphere projection on the image plane.

The second developed correction allows calculating the ellipse axes (this ellipse is the projection of the sphere on the image) from the obtained values in the horizontal and vertical lines with intersection in the ellipse centre (see Figure 6). In Figure 6, take note that *x_u1_* and *x_u2_* are not on the axis (the same for the points *y_u1_* and *y_u2_* their use thereby influences negatively in the determination of the sphere position (according to it is described in Section 5.2). The objective is to obtain the axes major and minor of the projected ellipse in the image (*a_e_*, *b_e_*) from the known information (*x_u1_* and *x_u2_*). Once the semi-minor axis of the projected ellipse is calculated, the algorithm seen in Section 5.2, in order to calculate the centre of the sphere definitively, is used.

Figure 6 shows the error due to projection deformation; it can be modelled by knowing the angle *θ_z_* and the object position along the axis *X_C_*. *θ_z_* is the angle between the optical axis and the line that passes through the centre of the ball and the centre of the projected ball. Note that *θ_z_* coincides with the angle by where the image plane can be rotated around the minor axis of the ellipse in order to project a circle on the plane image, thus:

(17) $$\theta_{z}=a\;\text{cos}\left(\frac{Z_{B}}{\sqrt{{X_{B}}^{2}+{Y_{B}}^{2}+{Z_{B}}^{2}}}\right)$$

where (*X_B_*, *Y_B_*, *Z_B_*) is the previously estimated position of the sphere centre in the camera coordinates system. On the other hand, the minor (*b_e_*) and major (*a_e_*) axes of the projected ellipse are related by:

(18) $$b_{e}=a_{e}\cos(\theta_{z})$$

**Figure 7.**
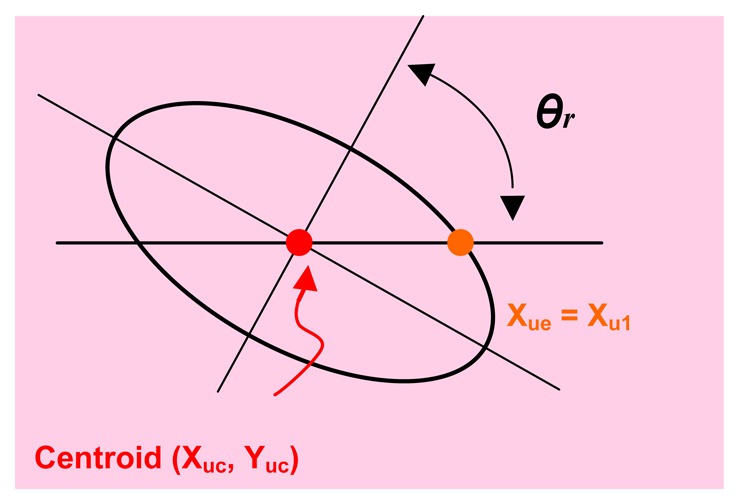
Angle *θ_r_*.

Observing Figure 6, it is deduced that the rotated ellipse is modelled suitably with the canonical parametric model. The projected ellipse in its rotated canonical parametric form can be expressed as:

(19) $$\begin{array}{c} x_{ue}=b_{e}\cos(\theta_{e})\cos(\theta_{r})-a_{e}\sin(\theta_{e})\sin(\theta_{r})+x_{uc}\\ y_{ue}=b_{e}\cos(\theta_{e})\sin(\theta_{r})-a_{e}\sin(\theta_{e})\cos(\theta_{r})+y_{uc} \end{array}$$

where *θ_e_* is the angle of a point in the canonical form of the ellipse, *θ_r_* is the angle by which the ellipse is rotated around the *Z* axis of the camera (Equation (19)) and (*x_uc_y_uc_*) is the centre of the ellipse in the image, Figure 7.

(20) $$\theta_{r}=\text{arctan}\left(-\frac{y_{uc}}{x_{uc}}\right)$$

The ellipse point in the ellipse horizontal line that pass by the centre satisfies (see Figure 7) *x_uc_* = *x_ue_* + (*x_u2_*−*x_u1_*)/2; *y_ue_* = *y_uc_*. By substituting in Equation (19) it is possible to obtain:

(21) $$\begin{array}{c} \left(x_{u2}-x_{u1}\right)/2=b_{e}co\text{s}(\theta_{e})\cos(\theta_{r})-a_{e}\text{sin}(\theta_{e})\text{sin}(\theta_{r})\\ 0=b_{e}\cos(\theta_{e})\sin(\theta_{r})+a_{e}\sin(\theta_{e})\cos(\theta_{r}) \end{array}$$

By substituting Equation (18) in (21) and clearing for *a*_e_ and *θ_e_* finally it is obtained:

(22) $$\begin{array}{c} \theta_{e}=\text{arctan}(-\tan(\theta_{r})\cos(\theta_{z}))\\ 0\;a_{e}=\left|\frac{\left(x_{u2}-x_{u1}\right)/2}{\cos(\theta_{z})\cos(\theta_{e})\cos(\theta_{r})-\text{sin}(\theta_{e})\text{sin}(\theta_{r})}\right| \end{array}$$

With the value of *a*_e_, *b*_e_ is calculated and setting in Equation (19) the values of $\theta_{e}=0,\overset{\pi }{\;}/\underset{2}{\;},\pi ,\overset{\pi }{\;}/\underset{2}{\;}$, allows us to determinate the new values for the extreme points used in the Equations (10) and (14); afterwards, the sphere centre position is calculated with the Equations (12) and (16). And so on. It could continue calculating with the new data this algorithm, but it has been checked that the improvement is not appreciable after a second iteration.

### 5.4. Summary of the Proposed Calibration Method

The proposed calibration algorithm is executed according to the following steps:

- Initialize the actuators of the robot in a home position (*θ_i0_*, that it is taken as unknown in the calibration algorithm).
- Randomly, inside of the workspace (not necessary well conditioned but near of the workspace boundaries) of the robot, generate a set of 300 poses of the robot to be used as calibration poses.
- The home of the robot is taken as unknown (*θ_i0_* and ***P****_0_*), but incremental measurements are acquired from: encoders of the actuators (incremental angles, *Θ_ik_*) and from the visual system (incremental poses, ***Π****_k_*).
  - Acquire 500 images in each pose of the robot (using the set in II) and by using image processing from each image *α_1_*, *α_2_*, *β_1_*, and *β_2_* are obtained. By using Equations (12) and (16) obtain (*X_B_*, *Y_B_*, *Z_B_*) is obtained.
  - In order to correct the projection of the spherical object in the image, use Equation (22) to correct (*X_B_*, *Y_B_*, *Z_B_*) and repeat above step once.
- Obtain new parameters of the robot by using above incremental measurements and equation in (8). It is well known that Newton-Raphson method could not converge to useful value. Thus result should be tested.

## 6. Results

A set of test cases is implemented to check the goodness of the parameters obtained by the calibration algorithm. The behaviour between a calibrated and a not calibrated robot system is compared. Two types of tests, static and tracking tests have been implemented. By executing static tests, it is determined how calibration parameters affect the robot positioning. By the second type, it is determined how calibration parameters affect the tracking tasks in which the RoboTenis has been designed.

### 6.1. Static Tests

**Figure 8.**
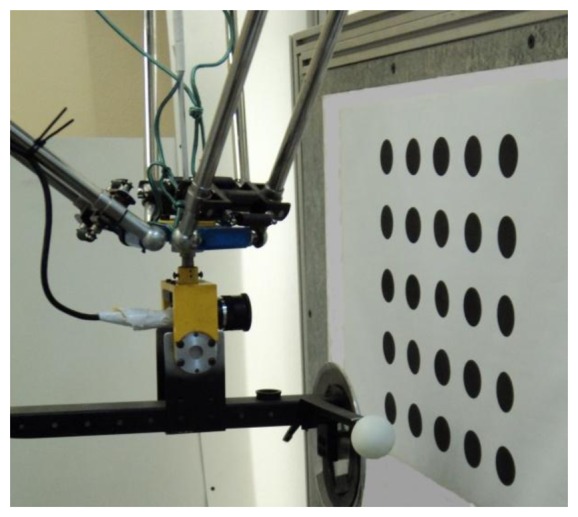
RoboTenis system and control element.

The objective of the static tests is to quantify the effect over the robot positioning by using the parameters obtained from the robot calibration process against the nominal parameters. Both parameter sets are shown in Table 1. Furthermore, the tests contribute significant information about the system behaviour. The test environment is made up of the RoboTenis (the system to test), a vision camera (measurement system) and a control element. Following the same idea in the robot calibration algorithm, the vision camera fixed to the robot hand in order to see the fixed objects in the robot visual area, is used as lonely measurement too. In order to simplify, the control element is two-dimensional formed by a plane surface with 25 black circles of 40 mm of diameter separated by 100 mm to each other, see Figure 8. A three-dimensional control element would have complicated the process without a significant information contribution. The circles have been printed on high quality and accuracy and the paper is glued to a rigid plate to make a plane exact. This control element is placed on vertical places in different distances from the robot and on a parallel plane to the image plane in each test, as see in Figure 8.

In order to evaluate the results in the robot motion, the data from the visual system when it sees the control element have been analyzed. Indeed, the repeatability of the three-dimensional data, when a circle to 200 mm of distance is seen by the camera in the image centre (to minimize the camera calibration errors) with the robot blocked, has also been analyzed. The visual resolution is 0.4 mm per pixel approximately in the horizontal axis. The circle position is determined by analysis of the circle rim with subpixel precision. Other results are the centre and diameter. A loop of 50 samples with the robot blocked allows the repeatability of the measurement to be obtained. Some representative data are shown in Table 2. It is important to emphasize the difference between X_c_ and Y_c_ coordinates (image plane), due to a different camera resolution and a bigger uncertainty in the camera Z_c_ axis since it depends on the circle diameter.

The high variation of the data shown in Table 2 means; it is not possible to use any visual measurement to estimate the robot position with high accuracy. In the static measurements, the mean of 500 samples of the circle is used because it is significantly more accurate. Standard deviation and Range for 25 measurements calculated one by one from 500 samples are shown in Table 3.

The repeatability of the measurement according to the ISO 9283 [41] (see Appendix) is 0.0635 mm. The data obtained with other test sets are similar.

The next element to be considered is the robot system itself. The maximum accuracy depends on the considered working point. With the used encoders, it is not possible to guarantee an accuracy that is less than 0.02 mm. In order to determine the robot position, the camera is placed 200 mm of distance. in front of each circle The robot analyzes the image and fixes its position till the circle centre matches the image centre, with an error less than 1 pixel (to minimize the camera calibration errors). The robot position data from the encoders are corrected with the vision system data. As previously described, in order to minimize the error in the visual data acquisition, a reliable measurement for the mean of 500 snapshots and simple data processes are considered. In such manner, accurate calculations of robot position with respect to the control element can be obtained.

#### 6.1.1. Positioning Repeatability Analysis

Only positioning repeatability has been analyzed as the first analysis. Position accuracy depends on the calibration parameters used. It is the main aim of this research and is analyzed in Section 6.1.2 with incremental data. Orientation repeatability and accuracy have not been analyzed because the robot system has only three degrees of freedom, all of them being translation.

According to the ISO 9283 protocol for positioning repeatability, a plane in a diagonal of a cube into the robot working area is chosen. In the plane, five points are chosen (the vertices of a rectangle and its centre). The robot has to execute a loop formed by positioning in front of each point and repeatable measure the last one (focus point). For such, as mentioned above, the control element is placed 200 mm with a black circle in the image centre. The visual system analyzes the three-dimensional position of the circle. After 500 samples, the position of the robot is known.

In Table 4, the obtained values are shown after the positioning loop in the five points, have been executed 25 times. The results are similar and do not depend on the focused point (central point results in Table 4). The tests have been done with two robot speeds, 1.0 m/s and 2.0 m/s, with a negligible load (camera and bat). Table 4 shows the range of the visual data in 25 samples and the repeatability calculated according to the ISO 9283 (see annex A). The data are coherent with the robot accuracy (0.02 mm) and uncertainty visual data (0.0635).

As conclusion, it can be affirmed that the robot system and the visual sensor have repeatability of less than 0.1 mm under normal working conditions (space working points, robot speed, and load). This value has to be quite small enough so as not to affect in the accuracy of the positioning distance (next section).

#### 6.1.2. Accuracy of the Positioning Distance Analysis

**Figure 9.**
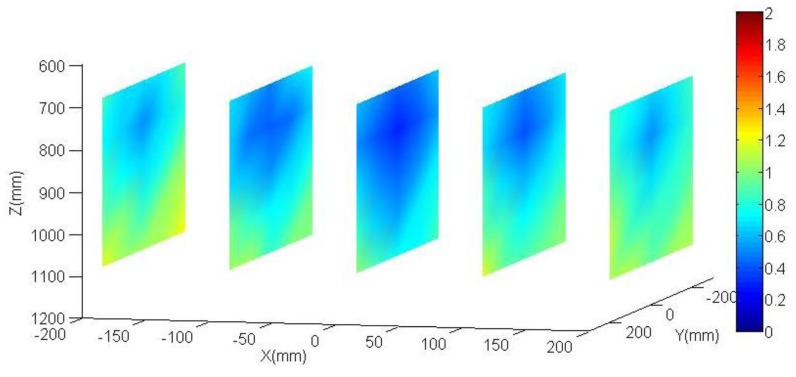
Spatial distribution of the obtained errors by using calibrated parameters. The mean of all errors is 0.583 mm (dimension values in mm).

**Figure 10.**
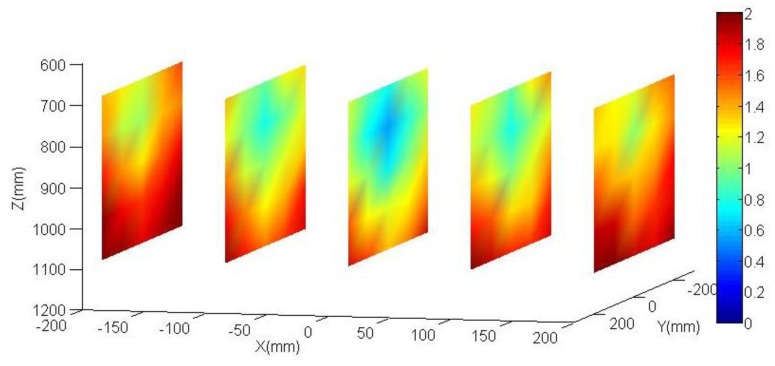
Spatial distribution of the obtained errors by using nominal parameters. The mean of all errors is 1.468 mm (dimension values in mm).

The aim of the following tests is to quantify the produced effect in the accuracy robot positioning by the parameters obtained in the calibration robot process against the nominal parameters. Both parameter sets are shown in Table 1. In order to obtain such, again, the vision system and control element have been used once more. Since no external sensor is used neither an external absolute reference system is needed, so it is more representative to analyze the accuracy of the positioning distance. The control system has been placed in five fixed vertical planes which take a homogeneous working robot area (see Figure 9 and Figure 10). Each plane is placed parallel to the camera plane, perfect parallelism is not required. The distance between the planes is 100 mm. It does not need to be perfectly positioned since the data to be analyzes are the distances between the circles of the same plane. The analyzed area corresponds to a cube of 400 mm in each side. This physical configuration allows studying the accuracy of the positioning distance as function of the position inside the working area.

The robot positioning in relation to the control element is done likewise as previously mentioned. The robot is placed 200 mm in front of each circle, the position is fixed till the circle is in the image centre, and the three-dimensional data position corrected with the visual information is stored. Its accuracy is considered well known to measure the mean of 500 samples.

For each circle to be analyzed (five planes with 25 circles each one), the distance between the circle and its neighbour in the same plane has been calculated by getting the absolute error. The absolute error is calculated by comparing the obtained distance with the theoretical distance of the control element. For this study a loop of 25 samples has been done according to the ISO 9283 (see Appendix). As there are several distances (so many as neighbours). The mean of the error in every distance has been calculated.

Figure 9 and Figure 10 show results obtained both for the calibrated parameters and the nominal parameters. The error is rendered by false colour, scale between 0 and 2 mm. For each one out of five planes distributed along the working area (in the XYZ coordinate system, located at the base of the robot, Figure 2), an interpolation has been done between the obtained results for each circle of the plane. Also the mean of all the errors is shown. As it can be observed, the errors obtained in the positioning distance decreased almost 40% using calibrated parameters. This justifies the efforts invested in the robot calibration. Among other reasons why it is not possible to minimize that error any farther, it is important to highlight that the method does not calibrate all robot parameters. It can be also observed that the errors increase in the working area limits.

### 6.2. Tracking Test

**Figure 11.**
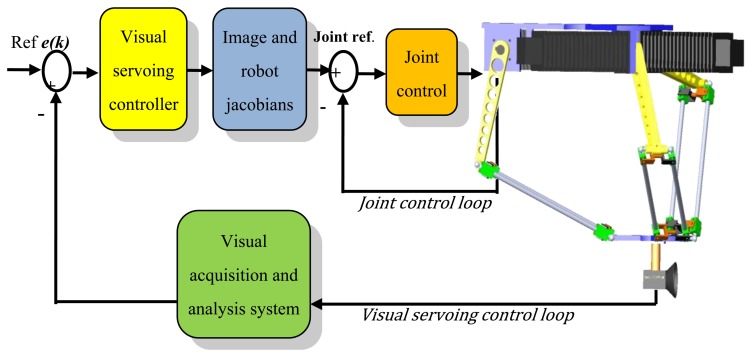
Visual servoing controller scheme of the RoboTenis System.

The objective of these tests is to quantify how calibration parameters affect the tracking tasks. It is desired that the robot is able to track an object that moves chaotically in its workspace. In order to test the calibration method, nominal parameters and calibrated parameters are compared when they are utilized in a position-based visual controller [42]. The robot and its visual controller are designed to carry out tracking tasks. For more information, please consult [43]. The visual controller is based on a well established architecture called: dynamic position-based look-and-move visual servoing. In this scheme (Figure 11), the visual controller makes use of image estimates (position and velocity), the Jacobian of the robot and its kinematic model in order to act over the robot actuators. Thus errors in the kinematic model generate undesirable movements that have an effect on the performance of the visual controller.

First this has been studied by simulating the effect it can have on tracking tasks using erroneous values of the parameters of the robot. It seeks to limit its maximum theoretical influence. Later follow-up tests are real, using both nominal and calibrated values.

#### 6.2.1. Simulation of the Influence of the Use of Incorrect Parameters

The visual control law based on position can be defined [42] as:

(23) $${\hat{u}}_{r}=-K_{p}e_{r}+{\hat{v}}_{p}$$

where:

*K_p_* is a positive scalar which fits experimentally (for test 16.8);
**e_r_** is the error in Cartesian coordinates, defined as the difference between the actual relative position of the object to the robot, and the desired relative position. It is obtained from visual features;
**v̂_p_** is the estimated speed of the monitored object.

If the direct Jacobian robot [26] is defined as:

(24) $$\dot{r}=J_{R}\dot{q}$$

where:

Articulate position:**q**=[*q*_1_*q*_2_*q*_3_]*^T^*
Cartesian position: **r**==[*X Y Z*]*^T^* (absolute position of the robot, Figure 2).

The RoboTenis system, like the Delta robot, has a Jacobian as a 3 × 3 matrix, which supports reverse except singular points.

The joint control law, which is acting on the engines, is:

(25) $${\hat{u}}_{q}={\hat{J}}_{R}^{-1}{\hat{u}}_{r}={\hat{J}}_{R}^{-1}\left(-K_{p}e_{r}+{\hat{v}}_{p}\right)$$

where **Ĵ_R_** is the estimated Jacobian, which can be obtained from or nominal or calibrated values. This Jacobian is the one used in the implementation.

The Cartesian robot behaviour (without considering the dynamics of the system) depends on the real robot Jacobian, which is unknown. It corresponds to what would move the robot when the motors are actuated.

(26) $$u_{r}=J_{R}{\hat{J}}_{R}^{-1}{\hat{u}}_{r}=J_{R}{\hat{J}}_{R}^{-1}\left(-K_{p}e_{r}+{\hat{v}}_{p}\right)$$

In order to analyze the influence of the use of nominal or calibrated parameters in the control law, it is possible to compare the difference in the movement of the robot between when using a control law with nominal parameters or when using calibrated. If defined:

**Ĵ**_RC_ Direct robot Jacobian obtained from calibrated parameters
**Ĵ**_RN_ Direct robot Jacobian obtained from nominal parameters
**J**_R_= **Ĵ**_RC_ Considered the best estimation

It is obtained:

(27) $$\Delta u_{r}=u_{rN}-u_{rC}={\hat{J}}_{RC}\left({\hat{J}}_{RN}^{-1}-{\hat{J}}_{RC}^{-1}\right)\left(-K_{p}e_{r}+{\hat{v}}_{p}\right)$$

**Figure 12.**
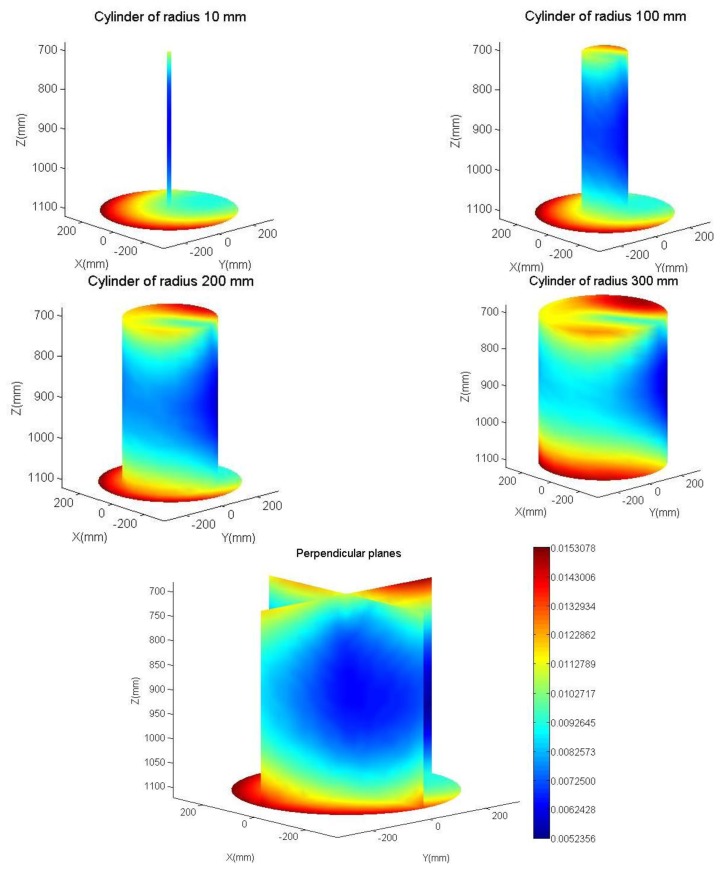
Largest singular value of the matrix Δ**Ĵ**_R_ in a cylinder of radius 300 mm and height 400 mm, included in the workspace.

Also:

(28) $$\Delta u_{r}={\hat{J}}_{RC}\left({\hat{J}}_{RN}^{-1}-{\hat{J}}_{RC}^{-1}\right)v=\left({\hat{J}}_{RC}{\hat{J}}_{RN}^{-1}-I\right)v$$

where **v** is a generic input speed. It is necessary to analyze the influence of the term $\Delta {\hat{J}}_{\text{R}}={\hat{J}}_{\text{RC}}({\hat{J}}_{\text{RN}}^{-1}-{\hat{J}}_{\text{RC}}^{-1})$ at different velocities. Figure 12 shows a spatial distribution of the largest singular value of said matrix into a cylinder of radius 300 mm and height 400 mm, included in the workspace. The absolute coordinates of the robot, (X, Y, Z), are used. The largest singular value is the spectral norm of the matrix and it represents, in that matrix, the upper limit of how the change speed influences in the control law.

For a better visualization, the largest singular value along the exterior of various cylinders (10 mm, 100 m, 200 mm, and 300 mm in radius), and in two perpendicular planes is represented in this figure with false colour. The last figure (on the right) shows the colorimetric scale used. The observed asymmetry is due to the lack of symmetry of the calibrated parameters and its difference from the nominal values. The simulation environment has avoided the singular points of both Jacobians, those points alter the study. The actual control avoids such singular points obtained using the Jacobian at a near no singular point.

Assuming maximum values:

‖**e**_r_‖=30 mm
‖**v̂**_p_‖=1500mm/s
‖**v**‖=16.8*30+1500≈2000mm/s
$\left\|{\hat{J}}_{\text{RC}}({\hat{J}}_{\text{RN}}^{-1}-{\hat{J}}_{\text{RC}}^{-1})\right\|=0.015$

It is obtained:

‖Δ**u_r_**‖≈0.015*2000≈30mm/s

It represents approximately 1.5% difference from the control law, which is the greatest influence that would be obtained by using the parameters calibrated against nominal values.

#### 6.2.2. Tracking Real Tests

In order to analyze the influence of a best calibration on the robot parameters, several tracking tests of a ball with a chaotic movement at speeds up to 1,500 mm/s have been done. The objective is to keep the robot at a constant distance of 600 mm with the ball in the image centre. In Table 5, the mean of the tracking errors in millimetres when 10 tests are done for the calibrated parameters and for the nominal parameters is shown, in function of the speed of the ball. Each test consists of 5,000 samples. A certain improvement, in a smaller proportion compared to the one indicated in Section 6.1.2, can be observed. This aspect can be explained by the high tolerance in the image characteristics-based control with the influence of the calibration errors.

## 7. Conclusions

In this paper, a calibration method using a camera attached to the robot hand as the only main sensor in order to improve the accuracy of a parallel robot inspired by the Delta robot is described. The simplicity of the equipment used over other more expensive alternatives, makes this proposal especially useful for calibration of robots in educational and research environments. The methodology for visual data acquisition is especially accurate, and can be extended to other applications.

The improvements using this method have been verified by executing several sets of tests (static and tracking tests). Before these tests, the measure repeatability of the whole system, which is close to 0.0635 mm according to [41], has been analyzed.

From the first static tests, it can be affirmed that the positioning repeatability of the robot system and the visual sensor is less than 0.1 mm under normal working conditions (space working points, robot speed, and load). From the second kind of static tests, it can be pointed out that using calibrated parameters reduces the errors in the positioning distance by almost 40%, which is a remarkable error reduction.

On the other hand, the tracking tests, by defining an index depending on the errors in the controller and an estimated working velocity, both sets (nominal and corrected) of kinematic parameters have been compared through the visual servoing controller performance. The increase in the performance obtained from the tracking visual controller regarding the correction of the parameters is extremely minimal (errors in the controller are reduced by more than 1.5% when the robot is calibrated). The improvements in the controller performance can be explained as follows: The image visual controller makes use of the Jacobian robot and kinematic robot models (inverse and direct). When the controller corrects the robot trajectory, errors in the model have a direct influence over the direction in which the controller tries to correct. On the other hand if the position of the object remains fixed, the error in the visual controller converges to zero but the evolution of the error is different for two set of parameters.

One aspect to analyze in future work is the incorporation of non-calibrated parameters in the optimization algorithm in order to further minimize the errors obtained. Some videos of the RoboTenis system in tracking tasks (such as playing ping-pong) are shown at: http://www.disam.upm.es/vision/projects/robotenis/indexI.html.

## Figures and Tables

**Table 1.** Robot kinematic parameters, nominal and calibrated.

	**Nominal**			**Calibrated**		

	i = 1	i = 2	i = 3	i = 1	i = 2	i = 3
*a_i_* (mm)	500	500	500	500.89	500.01	499.96
*b_i_* (mm)	1,000	1,000	1,000	1,002.86	1,001.37	1,001.28
*h_i_* (mm)	50	50	50	48.69	49.96	48.68
*θ_i_* (degrees)	0	0	0	0.316	0.844	0.936
***P****_x_* (mm)	0			5.31		
***P****_y_* (mm)	0			0.31		
***P****_z_* (mm)	750			743.74		
Φ_i_ (degrees)	**0**	**120**	**240**	**0**	**120**	**240**
H_i_ (mm)	**210**	**210**	**210**	**210**	**210**	**210**

Parameters that were not estimated.

**Table 2.** Standard deviation and Range (max value–min value) of 50 samples.

**Axis**	**Standard deviation**	**Range (mm)**
**X_c_**	0.014	0.059
**Y_c_**	0.038	0.163
**Z_c_**	0.092	0.497

**Table 3.** Standard deviation and Range for 25 measures analyzed.

**Axis**	**Standard Deviation**	**Range (mm)**
**X_c_**	0.0025	0.0096
**Y_c_**	0.0049	0.0150
**Z_c_**	0.0212	0.0846

**Table 4.** Repeatability of position, loop 25 poses.

**Speed (m/s)**	**Visual Data Range (X_c_, Y_c_, Z_c_) with 25 Samples (mm)**	**Repeatability of Position (mm)**
1.0	0.032, 0.025, 0.114	0.07041
2.0	0.039, 0.034, 0.121	0.07512

**Table 5.** Tracking Error Mean (mm).

**Maximum Speed of the Ball**	**Error with Nominal Parameters**	**Error with Calibrated Parameters**
500 mm/s	4.54	4.47
750 mm/s	7.42	7.32
1,000 mm/s	12.76	12.61
1,250 mm/s	20.14	19.84
1,500 mm/s	30.47	30.14

## Appendix

The following operation rules have been extracted from ISO 9283 (1998)

### Position Repeatability

The repeatability of position expresses the dispersion of the reached positions after n repeated visits in the same direction to a prefixed position.

Let *x_j_*, *y_j_*, *z_j_* be the coordinates of the jth reached position, and *x̄*, *ȳ*, *z̄* the barycentre coordinates of the set of obtained points after repeating the same position n times, according to:

$$\begin{array}{ccc} \bar{x}=\frac{1}{n}\overset{n}{\underset{j=1}{\text{∑}}}x_{j}; & \bar{y}=\frac{1}{n}\overset{n}{\underset{j=1}{\text{∑}}}y_{j}; & \bar{z}=\frac{1}{n}\overset{n}{\underset{j=1}{\text{∑}}}y_{j} \end{array}$$

The repeatability is defined as:

$$RP=\bar{l}+3S$$

where:

$$\begin{array}{ccc} \bar{l}=\frac{1}{n}\overset{n}{\underset{j=1}{\text{∑}}}l_{j} & \text{with} & l_{j}=\sqrt{{\left(x_{j}-\bar{x}\right)}^{2}+{\left(y_{j}-\bar{y}\right)}^{2}+{\left(z_{j}-\bar{z}\right)}^{2}} \end{array}$$

and:

$$S=\sqrt{\frac{\overset{n}{\underset{j=1}{\text{∑}}}{\left(l_{j}-\bar{l}\right)}^{2}}{n-1}}$$

### Positioning Distance Accuracy

Let *P_c1_*, *P_c2_* be the prefixed positions and *P_1,j_*, *P_2,j_* the reached positions after the j**^th^** repetition, the positioning distance accuracy is defined as the difference between the prefixed positions and the mean of the difference among the reached.

Let *x_c1_*, *y_c1_,z_c1_* and *x_c2_*, *y_c2_,z_c2_* be the coordinates of the two prefixed positions and *x_1j_*, *y_1j_,z_1j_* and *x_2j_*, *y_2j_,z_2j_* the coordinates obtained in the jth reached position in a set of *n* repetitions, then the positioning distance accuracy is calculated by using:

$$AD=\left|\bar{D}-D_{c}\right|$$

where:

$$\begin{array}{ccc} \bar{D}=\frac{1}{n}\overset{n}{\underset{j=1}{\text{∑}}}D_{j} & \text{con} & D_{j}=\left|P_{1j}-P_{2j}\right|=\sqrt{{\left(x_{1j}-x_{2j}\right)}^{2}+{\left(y_{1j}-y_{2j}\right)}^{2}+{\left(z_{1j}-z_{2j}\right)}^{2}} \end{array}$$

and:

$$D_{c}=\left|P_{c1}-P_{c2}\right|=\sqrt{{\left(x_{c1}-x_{c2}\right)}^{2}+{\left(y_{c1}-y_{c2}\right)}^{2}+{\left(z_{c1}-z_{c2}\right)}^{2}}$$
